# Predicting Crop Evapotranspiration by Integrating Ground and Remote Sensors with Air Temperature Forecasts †

**DOI:** 10.3390/s20061740

**Published:** 2020-03-20

**Authors:** Anna Pelosi, Paolo Villani, Salvatore Falanga Bolognesi, Giovanni Battista Chirico, Guido D’Urso

**Affiliations:** 1 Department of Civil Engineering, University of Salerno, 84084 Fisciano, Italy; p.villani@unisa.it; 2 Interdepartmental Research Centre on the "Earth Critical Zone" (CRISP) of the University of Naples Federico II, 80055 Portici, Italy; durso@unina.it; 3 Ariespace s.r.l., 80143 Naples, Italy; salvatore.falanga@ariespace.com; 4 Department of Agricultural Sciences, University of Naples Federico II, 80055 Portici, Italy; gchirico@unina.it

**Keywords:** crop evapotranspiration, remote sensing, numerical weather forecasts, Hargreaves–Samani equation, Penman–Monteith equation, irrigation advisory systems

## Abstract

Water use efficiency in agriculture can be improved by implementing advisory systems that support on-farm irrigation scheduling, with reliable forecasts of the actual crop water requirements, where crop evapotranspiration (ET_c_) is the main component. The development of such advisory systems is highly dependent upon the availability of timely updated crop canopy parameters and weather forecasts several days in advance, at low operational costs. This study presents a methodology for forecasting ET_c_, based on crop parameters retrieved from multispectral images, data from ground weather sensors, and air temperature forecasts. Crop multispectral images are freely provided by recent satellite missions, with high spatial and temporal resolutions. Meteorological services broadcast air temperature forecasts with lead times of several days, at no subscription costs, and with high accuracy. The performance of the proposed methodology was applied at 18 sites of the Campania region in Italy, by exploiting the data of intensive field campaigns in the years 2014–2015. ET_c_ measurements were forecast with a median bias of 0.2 mm, and a median root mean square error (RMSE) of 0.75 mm at the first day of forecast. At the 5^th^ day of accumulated forecast, the median bias and RMSE become 1 mm and 2.75 mm, respectively. The forecast performances were proved to be as accurate and as precise as those provided with a complete set of forecasted weather variables.

## 1. Introduction

Optimal irrigation water management is one of the main challenges of precision agriculture, especially in open field crops, where farmers must deal with the uncertainty of both weather conditions and water availability, under climate change scenarios [1]. In many regions of the world, such as Mediterranean areas, where open field irrigation is practiced during dry seasons characterized by negligible rain contributions, irrigation water requirements are essentially driven by crop evapotranspiration. 

The evapotranspiration of crops under standard conditions, i.e., the evapotranspiration of crops grown in large fields under excellent agronomic and soil water conditions (hereinafter referred to as ET_c_), according to FAO Irrigation and drainage paper n. 56 [2], can be estimated by models, such as the Penman–Monteith equation, that require data reflecting the crop canopy properties and ground surface weather conditions. The availability of these data at reasonable costs represents a prerequisite for the implementation of these models within operational advisory systems, that can help farmers in assessing the actual crop water requirements, and thus optimizing irrigation scheduling. 

Some Earth observation (EO) systems provide free multispectral imagery of the crop, that can be exploited for retrieving the parameters of the crop canopy influencing the evapotranspiration processes [3]. Assessing crop canopy parameters from multispectral imagery still represents a groundbreaking and active research topic. The newest satellite missions provide multispectral images with high spatial and temporal resolutions, which enhance the reliability of the remotely-assessed crop parameters [4]. Currently, the National Aeronautics and Space Administration (NASA) and the European Space Agency (ESA), the main world space agencies, offer freely available georeferenced multispectral imagery with a high spatial resolution (30 m or less) and short time intervals (9 days or less), by means of dedicated web platforms. Programs like Landsat 8 by NASA and Sentinel-2 by ESA, by offering free access to the satellite images, have prompted the development of agricultural advisory services based on the remote assessment of the crop canopy properties and evapotranspiration. Examples of irrigation advisory services based on remote multispectral images are IRRISAT in Southern Italy, EO4Water in Lower Austria, and IRRIEYE in Southern Australia [5].

Ground weather conditions can be efficiently monitored by ground weather stations. However, farmers and irrigation system operators require crop water requirement estimates a few days in advance for optimal irrigation scheduling [6]. Thus, the most advanced advisory services, such as IRRISAT in Southern Italy [5], rely on weather forecasts produced by meteorological services, that implement NWP models both at regional and global scales. 

The reliability of weather outputs produced by NWP models has considerably improved in the 21st century [7]. This circumstance makes NWP model outputs a valuable source for estimating the meteorological variables relevant for calculating ET_c_ maps, as an alternative to the spatial interpolation of coarse, ground-based weather data [8]. Moreover, operational NWP model outputs can be exploited for forecasting ET_c_ a few days ahead, and thus optimize the irrigation schedule in real time [9,10,11].

When NWP outputs are exploited for forecasting evapotranspiration, the use of simplified ET_c_ estimation methods, which employ less weather variables than the Penman–Monteith equation [2], could be a good alternative to the Penman–Monteith equation for mainly two reasons:

(i) A smaller number of forecasted variables also means less sources of uncertainties, thus making the forecasts more robust [10];
(ii) The cost of advanced NWP outputs in operational mode is generally proportional to the number of weather variables required.

The first point (i) arises from the consideration that the forecasts of weather variables provided by NWP are subjected to errors, which differently affect the uncertainty of the forecasted ET_c_ [9,10]. NWP model errors in a generic point of the model grid are both systematic and non-systematic. These errors are generated by the model limitations in simulating the physical and dynamical properties of the system at the model resolution scale [12]. Generally, prognostic variables (i.e., those variables that are directly predicted by the numerical integration of the dynamics forcing equations that simulate the physics of the atmosphere, such as surface temperature) are less affected by errors than diagnostic variables (e.g., incident solar radiation and relative humidity), which are derived from NPW model prognostic variables. In addition, when NWP model outputs are compared with point observations, additional systematic and non-systematic model errors emerge. These additional errors are due to variabilities of the weather variables at spatial scales smaller than those resolved by the numerical grid [13,14]. However, NWP forecast errors can be reduced at the locations where data from ground weather sensors are available by statistical post-processing. 

Post-processing procedures based on a simple bias correction approach are particularly successful in improving the forecast performance of prognostic variables, such as temperature, at locations affected by systematic errors [15].

The second point (ii) descends from the consideration that real time forecasts of the weather variables required for ET_c_ forecasting according to the Penman–Monteith equation, are available only by subscribing costly services with the weather forecast providers. Public (and free) numerical data of weather forecasts are generally restricted to air temperature [16], while other weather conditions are generally described in a qualitative manner (e.g., weather type), or according to predefined scale (e.g., Beaufort scale of wind force). 

This study, as an extension of what had been anticipated in a previous conference paper [17], presents a detailed description and evaluation of a new methodology designed for forecasting the ET_c_ of open field crops, by integrating crop parameters data retrieved from freely available satellite multispectral imagery, weather data provided by ground sensors, and temperature forecasts. The performance of the proposed methodology is evaluated in the Campania region, Southern Italy.

## 2. Materials and Methods

### 2.1. Study Area and Data from Ground Sensors

**Figure 1:**
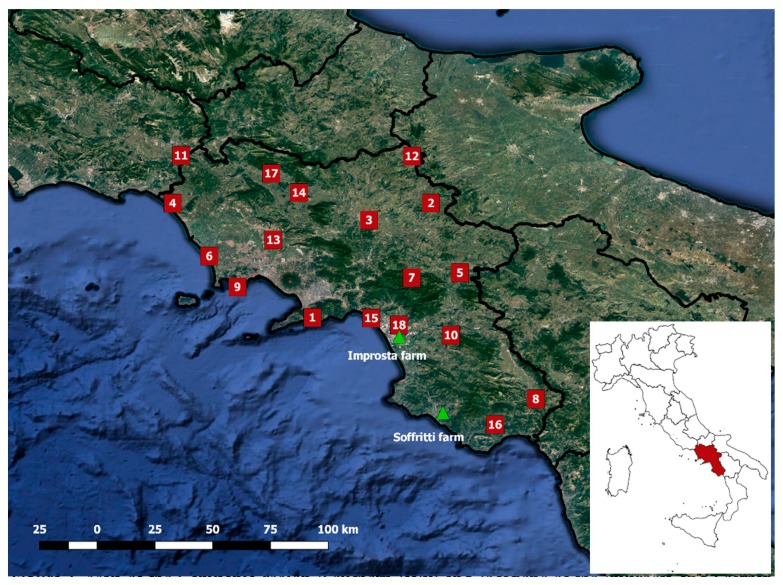
Map of the Campania region (Southern Italy) and locations of the Improsta and Soffritti farms (green triangles) and ground automatic weather stations (red squares).

The study area is the Campania region located in Southern Italy, as shown in Figure 1. The region has a surface area of about 14,000 km^2^, and extends between the Tyrrhenian Sea and the Southern Apennines. Most of the region is characterized by dry-summer, subtropical climates, with hot and dry summers. The mean monthly temperature ranges from 20 °C to 30 °C in summer, and between 5 °C and 15 °C in winter. The maximum monthly precipitation values are recorded during November and December, the minimum values during July and August. The spatial variability of the weather conditions is highly affected by the complex orography of the region [18].

Irrigation in open fields does not start before April, frequently in June, and it lasts until the end of September. However, the actual time range of the irrigation season is determined by the weather fluctuations and specific agricultural practices: in the following, we assume an irrigation season for the analyzed study cases from June to September.

In this region, two sets of data have been combined in order to achieve a representative ensemble of cases for evaluating the performances of the ET_c_ forecasted with the proposed methodology:

- Remotely-assessed canopy parameters of maize crop fields belonging to two farms, called Improsta and Soffritti;
- Weather observations and forecasts at 18 stations distributed across the Campania region.

The locations of the two farms and 18 weather stations are depicted in Figure 1.

The combined use of these two sets of data for evaluating the performance of the proposed methodology in forecasting ET_c_ is formally legit, since the temporal pattern of the canopy parameters, even though these can change between the different locations due to changes in local weather conditions across the Campania region, are statistically independent of the local NWP errors, and thus of the simulated ET_c_ forecasts.

The two farms Improsta and Soffritti are representative of two different topographic conditions: The Improsta farm is in a large floodplain; the Soffritti farm is in a hilly inland area (Figure 1). 

Two important field campaigns were conducted during the irrigation seasons from June to September 2014, in seven fields at the Improsta farm, and from June to September 2015 in six fields at the Soffritti farm. Ground-based measurements of the leaf area index (LAI), i.e., the one-sided green leaf area per unit ground surface area, were obtained with a LICOR LAI-2000 Plant Canopy Analyzer, and were used to validate the LAI retrieved from multispectral satellite imagery [6]. Ground validation of satellite LAI estimates are fundamental in areas with complex terrain features, which highly affect the quality of the multispectral satellite imagery [4]. 

The 18 ground-based automatic weather stations (AWS) have been operating since 2008. These stations are managed by the Regional Meteorological Service, and obey the EUMETNET technical specifications [19]: each station is equipped with two redundant sensors to provide measurements with high accuracy and precision standards. The AWS sites were chosen to achieve a good representation of the weather variability within the region, including both the coastal areas and the inland side of the region. The available data from these ground AWSs are air temperature and humidity at 2 m; global incoming solar radiation; wind speed at 10 m; barometric pressure and precipitation (that, for the scope of this study, has been neglected). All data have a time resolution of 10 minutes, and are then gathered in average daily values.

These sites, characterized by significantly different terrain conditions (from coastal to internal areas and from floodplains to hilly and mountain areas), are also relevant for having a representative evaluation of the NWP performances applied to ET_c_ forecasts. In fact, weather forecasts performances may vary even within such a small region as Campania, because NWP model resolutions cannot resolve the small-scale spatial variability of the weather conditions, especially in areas characterized by complex terrain features [13].

Table 1 presents the geographical coordinates of the AWS locations along with their elevations, ranging from 1 m a.s.l. to 848 m a.s.l., and the mean values of the weather data of interest for estimating ET_c_ with Penman–Monteith equation, registered from June to September in the years 2010–2015. It is interesting to observe that wind speed exhibits a high variability across the region, with values ranging from 1.10 ms^−1^ to 4.42 ms^−1^. This large variability of the wind speed reflects the impact of the local morphology. The highest wind speed values are recorded by those AWS (n. 5, 10 and 12) located close to the ridges of the Southern Apennines, exposed to winds blowing between the west and the eastern slopes. Average temperature ranges from 20.2 °C and 26 °C, with a variability mainly influenced by the distance from the Tyrrhenian seacoast and the elevation. The average relative humidity and solar radiation do not exhibit specific spatial patterns, being rather influenced by site-specific conditions.

### 2.2. Numerical Weather Forecasts

Forecasts of atmospheric pressure, solar radiation, wind speed at 10 m, relative humidity and temperature at 2 m were acquired from COSMO-LEPS outputs, with lead times from 1 to 5 days. 

COSMO-LEPS is a limited area ensemble prediction system, developed within the Consortium for small–scale modeling (COSMO), and operated by the HydroMeteoClimate Regional Service of Emilia–Romagna, Italy (ARPA–SIMC) with 16 (that now are 20, since 2016) members, and a spatial resolution of 7.5 km.

The study in [10] firstly showed that COSMO-LEPS produces skillful and reliable forecasts up to 5 days of the weather variables relevant for ET_c_ estimates in the Campania region. Moreover, it showed that the forecast performances do not significantly reduce by increasing the forecast lead time.

In this study, the numerical weather forecasts at the 18 AWS sites have been produced by a triangle-based, bi-linear interpolation method, which consists of interpolating the three numerical grid points closest to the examined AWS site. Then, the median value of the ensemble forecasts was computed for each AWS site. Since the COSMO-LEPS numerical outputs have a temporal resolution of three hours, the daily values of the forecasted variables have been calculated as the average values of the eight available forecasts produced for each day. 

COSMO-LEPS outputs, as all numerical weather forecasts, exhibit systematic and non-systematic errors when compared with local weather observations. These forecast errors, originating from the inability of the NWP model to represent the atmospheric processes at the sub-grid scales, can be reduced by using different statistical post-processing techniques when the weather data observed at ground AWSs are available [20]. 

Here, a simple bias correction of weather forecasts has been performed to improve local forecasts. The bias correction technique consists in correcting the forecasts by the mean bias computed in the reference period [21]. The mean bias to be removed from the forecasted weather variables in the irrigation seasons 2014 and 2015 was estimated by comparing COSMO-LEPS forecasts and ground weather data at each AWSs during the irrigation seasons (June–September) of the years 2010–2013. Then, the bias-corrected temperature forecasts were obtained by subtracting these mean biases from the original temperature forecasts. 

Only temperature forecasts were bias corrected, since temperature was the only forecasted weather variable affected by systematic errors that could be effectively removed by the bias correction at each AWS site. The application of the bias correction to the other forecasted weather variables produced contrasting results across the study region. Although an average slight increase of the performance was observed across the region, at some locations the forecast performance decreased, due to the presence of non-systematic errors in areas where the topographic conditions limit the NWP model performances.

### 2.3. Remote Sensors and Multispectral Images for Retrieving Crop Parameters

Maize canopies at both the Improsta and Soffritti fields have been monitored by processing multispectral images, provided by remote sensors operating on DEIMOS-1 and LANDSAT 8 satellites. Images from Sentinel-2 satellites were not available for the period to which this study refers. 

The crop canopy parameters used for estimating crop evapotranspiration with the Penman–Monteith equations are the albedo and the Leaf Area Index (LAI).

Albedo was used for estimating the fraction of incoming short-wave radiation leading to net radiation (*R_n_*) in the Penman–Monteith equation, and it represents an estimate of the hemispheric and spectrally integrated surface albedo. 

LAI was exploited for assessing the aerodynamic and surface resistances that appear in the Penman–Monteith equation [2]. 

LAI and albedo maps at Improsta Farm were retrieved by processing DEIMOS-1 multispectral satellite images with a spatial resolution of 22 m, and LANDSAT 8 multispectral images with a spatial resolution of 30 m, to obtain data with an average time frequency of 1 week, from June to September 2014. LAI and albedo maps at Soffritti Farm were derived from DEIMOS-1 and LANDSAT 8 multispectral imagery with a time frequency of 15 days, from June to September 2015.

DEIMOS-1 is a commercial tasking EO satellite, and is part of the Disaster Monitoring Constellation (DMC) (http://www.dmcii.com). The sensor records radiance in three spectral bands, corresponding to green (520–600 nm), red (630–690 nm) and near infrared (770–890 nm) parts of the electromagnetic spectrum at a ground sampling distance (GSD) of 22 m. DEIMOS-1 images were processed to a high degree of accuracy using an industry standard atmospheric correction algorithm (ATCOR-2) [22,23]. LANDSAT 8 OLI data at a GSD of 30 m were generated routinely by the Landsat Ecosystem Disturbance Adaptive Processing System (LEDAPS), and obtained through the United States Geological Survey (USGS) Land Science Research and Development (LSRD) website; LEDAPS uses Dense Dark Vegetation (DDV) targets to estimate the aerosol optical thickness (AOT). The estimated AOT was used afterwards as input to the Second Simulation of a Satellite Signal in the Solar Spectrum (6S) radiative transfer model. 

Taking into account the minimal spectral resolution of the EO data normally available, the albedo (*α*) was calculated as a weighted total of the spectral surface reflectance ($\rho_{bi}$) for a given band bias resulting from atmospheric correction with broadband coefficients ($\omega_{bi}$), reflecting the corresponding fraction of the solar irradiance in each sensor band [24]:

(1) $$\alpha =\sum_{bi}(\rho_{bi}\omega_{bi})$$

LAI was retrieved from the multispectral by using the CLAIR model. The CLAIR model assumes an inverse exponential function between LAI and the weighted differential vegetation index (WDVI) [25,26]. The WDVI is calculated by means of the reflectance observed in the remote images in red and near-infrared bands.

LAI was then calculated as follows:

(2) $$LAI=-\frac{1}{\alpha^{*}}ln\left(1-\frac{WDVI}{WDVI_{\infty }}\right)$$

where WDVI_∞_ is the asymptotically limiting value for the WDVI, while b is an extinction and scattering coefficient. 

The two CLAIR model parameters (WDVI_∞_ and the α* coefficient) can be identified by using chronologically paired LAI field measurements and reflectance values from satellite images [27]. In this study, LAI was computed by applying α* = 0.35, a value that was verified for this area from previous field campaigns [26]. 

### 2.4. FAO 56 Reference Evapotranspiration

The reference evapotranspiration is the evapotranspiration under standard conditions of a hypothetical grass reference crop, according to FAO Irrigation and drainage paper n. 56 [2]. It only depends on the weather data, and it was introduced to describe the evaporative demand of the atmosphere [2]. In this study, daily reference evapotranspiration was computed by applying daily weather data retrieved from ground sensors at the AWS sites. Hereinafter, we use the symbol ET_0-PM,obs_ to specify that reference evapotranspiration is computed with ground-based weather data, rather than numerical weather forecasts. ET_0-PM,obs_ (mm day^−1^) is computed as follows:

(3) $${\mathrm{ET}}_{0\text{-}\mathrm{PM},\mathrm{obs}}=\frac{1}{\lambda }\frac{\Delta \left(R_{n}-G\right)+\gamma \frac{900}{T+273}WS\left(e_{s}-e_{a}\right)}{\Delta +\gamma \left(1+0.34WS\right)}$$

where λ is the latent heat of vaporization, *e_s_* is the saturation vapor pressure (kPa), *e_a_* is the actual vapor pressure (kPa), Δ is the slope of the vapor pressure curve (kPa °C^−1^), and γ is the psychometric constant (kPa °C^−1^). *T* is the daily mean air temperature at 2 m height (°C). *WS* is the wind speed at 2 m height above ground (m s^−1^), that can be computed from the wind speed at 10 m above the ground by employing the logarithmic equation of the wind speed profile suggested by Allen et al. [2], that gives a multiplying factor of about 0.75. *R_n_* is the net radiation at the crop surface (MJ m^−2^ day^−1^). and *G* is the soil heat flux density (MJ m^−2^ day^−1^). The net radiation (*R_n_*) was calculated as the difference between the incoming net shortwave radiation and the outgoing net long-wave radiation. As suggested by Allen et al. [2], for the reference crop, the incoming net shortwave radiation is a fraction of the incoming shortwave solar radiation (*RS*), by setting the albedo equal to 0.23. Daily mean air temperature (*T*) was computed as the average of daily maximum and minimum air temperature computed at the highest available temporal resolution of data. The actual vapor pressure was computed as a function of the mean air relative humidity (*RH*).

### 2.5. Hargreaves–Samani Equation for Estimating Reference Evapotranspiration 

The Hargreaves–Samani equation was recommended as simplified method for estimating the reference evapotranspiration [2] for those sites where the availability of reliable weather data is questionable. 

Since Hargreaves–Samani is an empirical equation, its prediction performance varies according to the climatic characteristics of the region where it is used. Thus, its local calibration is recommended to better estimate ET_0_ [2]. Past studies suggested different techniques for calibrating the Hargreaves–Samani equation. The most common approach consists in calibrating a scaling coefficient [2,28].

Hargreaves–Samani referenced evapotranspiration is thus defined according to the following expression:

(4) $${\mathrm{ET}}_{0\text{-}\mathrm{HS}}=k_{HS}0.0023\left(T+17.8\right)\sqrt{T_{\max}-T_{\min}}\left(0.408R_{a}\right)$$

In Equation (4), ET_0-HS_ is the Hargreaves–Samani reference evapotranspiration in (mm day^−1^), *R_a_* is the extraterrestrial radiation (MJ m^−2^ day^−1^), *T_max_* and *T_min_* are respectively the daily maximum and minimum temperature (°C). The parameter *k_HS_* is the scaling factor to be calibrated. 

Hereinafter, we use the symbol ET_0-HS,obs_ when Equation (4) is applied with air temperature retrieved from ground sensors, while ET_0-HS_ is computed with forecasted air temperature. 

The scaling factor *k_HS_* is calibrated by minimizing the squared differences (ET_0-HS,obs_ − ET_0-PM,obs_)^2^ summed over the entire calibration period. In this study, *k_HS_* was locally calibrated at each AWS by using past ground sensors weather data during the irrigation seasons (June, 1st–September, 30th) in the period 2010–2013. The calibrated Hargreaves–Samani equation was then validated in the two following irrigation seasons of the years 2014–2015.

### 2.6. Application of the Penman–Monteith Equation with Remotely Assessed Crop Parameters

Following the remote sensing-based Penman–Monteith direct approach (also known as the one-step approach, [3]), the Penman–Monteith equation was employed for estimating the crop potential evapotranspiration under standard conditions as follows:

(5) $${\mathrm{ET}}_{c\text{-}\mathrm{PM}}=\frac{86400}{\lambda }\left[\frac{\Delta \left(R_{n}-G\right)+\rho c_{p}^{}\left(e_{s}-e_{a}\right)/r_{a}}{\Delta +\gamma \left(1+r_{s}/r_{a}\right)}\right]$$

where ρ is the mean air density at constant pressure (kg m^−3^), *c_p_* is the specific heat of the air (MJ kg^−1^ °C^−1^), *r_a_* and *r_s_* are respectively the aerodynamic and surface resistances (s m^−1^).

The weather data in Equation (5) can be either derived by ground sensors or by weather forecasts. Herein after we use the symbol ET_c-PM,obs_ to indicate the crop evapotranspiration estimated according to the Equation (5) with observed weather variables, reserving the ET_c-PM_ when Equation (5) is applied with forecasted weather variables.

The aerodynamic resistance is defined as follows:

(6) ra=ln[2−23hc0.123hc]ln[2−23hc0.0123hc](0.41)2WS

where *h_c_* is the crop height, that was set equal to 0.4 m, as common practice. It has been evaluated that the sensitivity of ET_c_ estimates to crop height in the range 0.1–0.6 m is negligible [25].

The surface resistance was computed as follows:

(7) $$r_{s}=\left\{ \begin{array}{l} \frac{200}{LAI}\;\forall LAI\leq 4\\ 50\;\;\forall LAI>4 \end{array}\right.$$

where LAI was estimated from remotely-sensed crop images.

The fraction of incoming short-wave radiation contributing to the net radiation *R_n_* was computed by multiplying the incoming short-wave radiation by (1-α), where α is albedo obtained by processing the satellite multispectral images. 

The Albedo and LAI in Equations (5)–(7) were updated, as a new multispectral crop image has been processed, and they were kept constant until the next satellite observation becomes available, as done in most operational advisory services [6]. Crop growth models could be applied to predict the time variation of crop parameters between consecutive satellite passages, but this is beyond the scope of this paper.

### 2.7. Crop Coefficient

The crop coefficient, K_c_, is a dimensionless coefficient that is defined as the ratio between the evapotranspiration of the examined field crop under standard conditions and the reference crop evapotranspiration.

Here, we were interested in analytically computing the crop coefficient K_c_ as follows:

(8) $$K_{c}=\frac{{\mathrm{ET}}_{c\text{-}\mathrm{PM},\mathrm{obs}}}{{\mathrm{ET}}_{0\text{-}\mathrm{PM},\mathrm{obs}}}$$

where ET_0-PM,obs_ and ET_c-PM,obs_, respectively, refer to the reference and the crop evapotranspiration computed with the Penman–Monteith equation with ground-measured weather data. ET_c-PM,obs_ was computed with Equations (5)–(7), with the crop canopy parameters LAI and albedo estimated from multispectral satellite images. 

The crop coefficient, as defined by Equation (8), depends on both crop parameters and weather data. In the proposed methodology, crop parameters in *K_c_* are updated as a new crop canopy image becomes available (almost weekly), while weather data are daily updated based on ground weather sensors.

### 2.8. Forecasting Crop Potential Evapotranspiration with Hargreaves–Samani Equation

In the proposed methodology, ET_c_ forecasts are estimated for lead times Δ*t* from 1 to 5 days by employing the Hargreaves–Samani equation (Equation (4)) with the forecasted bias-corrected temperature (instead of the observed temperature), multiplied by *K_c_* updated at the time *t_0_* of forecast delivery:

(9) $${\mathrm{ET}}_{c\text{-}\mathrm{HS}}\left(t_{0}+\Delta t\right)=K_{c}\left(t_{0}\right){\mathrm{ET}}_{0\text{-}\mathrm{HS}}\left(t_{0}+\Delta t\right)$$

For the sake of comparison, crop potential evapotranspiration has been also computed with the one-step approach by means of the Penman–Monteith ET_c-PM_ applied with the forecast variables, as done by Chirico et al. [6].

**Figure 2:**
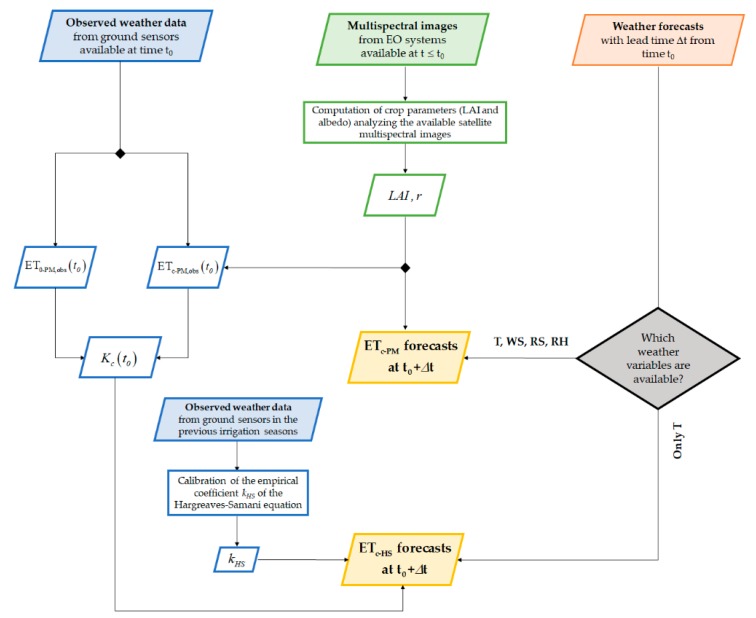
Flowchart providing an overview of the methodology for assessing ET_c_ forecasts by means of air temperature forecasts. The flowchart also illustrates the methodology for ET_c_ forecasting by using a complete set of forecasted variables, as required by the Penman–Monteith equation.

Figure 2 shows a flowchart that provides an overview of the proposed methodology. The input data sources are on the top of the diagram: (i) observed weather data, (ii) multispectral images and (iii) weather forecasts Our methodology intends to provide ET_c-HS_ forecast, by exploiting only temperature forecasts. The flowchart also illustrates the methodology for ET_c_ forecasting based on Penman–Monteith, thus exploiting a complete set of weather variables to be acquired from meteorological services.

### 2.9. Evaluation of Forecast Performances

Forecasts performances of the individual weather variables, as well as of ET_c-PM_ and ET_c-HS_ forecasts, have been evaluated by comparing them with the corresponding variables retrieved from ground sensors. For what concerns the evapotranspiration, ET_c-PM_ and ET_c-HS_ forecasts are compared with ET_c-PM,obs_, hereinafter also denoted as “best estimate”.

Statistical performance indices were computed for all lead times by comparing the median value of the ensemble of the forecasts on the generic i-th day, with the best estimate at the same day. The first index is the BIAS, which was used as an indicator of the accuracy of the forecasts:

(10) $$\mathrm{BIAS}=\frac{\sum_{i=1}^{n}\left(X_{f}^{i}\;-\;X_{\mathrm{obs}}^{i}\right)}{n}$$

where $X_{f}^{i}$ and $X_{obs}^{i}$, respectively, are the variables forecasted and observed at the i-th day, while *n* denotes the number of examined days, that in this study is 122 (from June, 1 to September, 30). 

The second performance indicator is the root mean square error, RMSE, which gives insight into both the accuracy and precision of the forecasts. RMSE is calculated as follows:

(11) $$\mathrm{RMSE}=\sqrt{\frac{\sum_{i=1}^{n}{\left(X_{f}^{i}\;-\;X_{\mathrm{obs}}^{i}\right)}^{2}}{n}}$$

where the symbols are the same as those used in Equation (10).

Forecast performances were evaluated at different lead times, i.e., from one day up to the five days after the weather forecasts were issued. The forecast performance of the evapotranspiration was evaluated by computing the corresponding values accumulated up to one, three and five days after the weather forecasts were issued.

## 3. Results

### 3.1. Weather Forecast Performances

**Figure 3:**
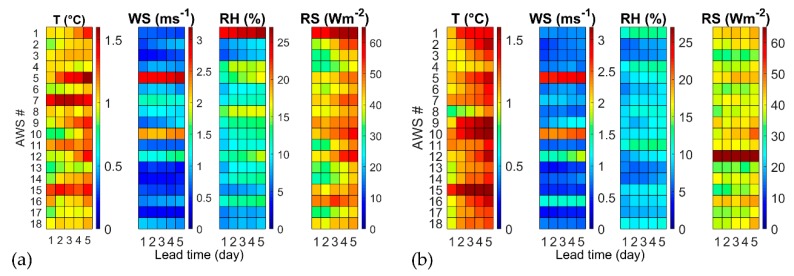
Root mean squared error (RMSE) of forecasted daily weather variables at the 18 automatic weather stations (AWS) sites for lead times from 1 to 5 days in the period: (**a**) June–September 2014; (**b**) June–September 2015.

The RMSE of the forecasted weather variables was computed to better understand the performance of the evapotranspiration predicted with the Hargreaves–Samani and Penman–Monteith equations. Temperature forecasts were corrected with a simple bias correction technique to remove the systematic bias. The systematic bias was estimated in the months between June and September of the years 2010–2013 (reference period). The average (among all sites and lead times) mean bias of the raw temperature forecasts in the reference period is about −0.68 °C. The mean bias is always negative by varying site and lead time, thus denoting a systematic underestimation of the observed temperature by the forecast outputs of COSMO-LEPS. The underestimation was corrected by subtracting the mean bias from the raw temperature forecasts at each AWSs and for each lead time. This correction led to a significant reduction of the mean bias (almost equal to zero), related to the corrected temperature forecasts and of the RMSE from an average value of 1.70 °C to 1.16 °C (as better detailed for each site, lead time and year in Figure 3). The bias correction was not applied to the other variables, since it produced contrasting results across the region. The colored grids in Figure 3 depict the RMSE values calculated at the 18 AWS sites (along the rows) for five forecast lead times (from 1 to 5 days). The RMSE value is represented according to the color scale on the side: blue corresponds to the minimum RMSE; dark red to the maximum RMSE. 

The forecast performance considerably changes across the region, but also from year 2014 to year 2015. Summer 2015 was characterized by anomalous unstable meteorological conditions, with colder temperature and less solar radiation. Temperature (T) RMSE is higher at sites like AWS n. 5–7 with irregular topography and at AWS n. 15 close to the coastline. Temperature RMSE is generally higher in 2015 than in 2014. Wind speed (WS) RMSE is generally below 1 ms^−1^, except for some windy locations (AWS n. 5, n. 10 and n. 12), with minor differences between 2014 and 2015. Air relative humidity (RH) is higher in 2014 than in 2015, except for the anomalous RMSE above 20% observed at AWS n. 1 in year 2014, while other stations exhibit their RMSE generally lower than 15%. RS RMSE is higher in 2014 than in 2015, except for AWS n. 12, that exhibited a RMSE larger than 60 W m^−2^. 

**Figure 4:**
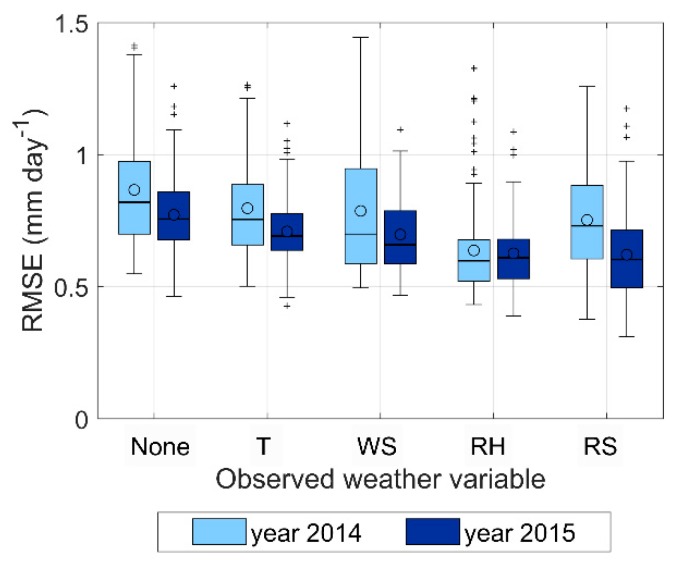
Impact of the forecast error of the single weather variable on the forecasted ET_c-PM_. The x axis indicates which weather forecast variable has been replaced by the corresponding observed variable for the RMSE assessment. RMSE is averaged over the five lead times. The spread of the box plot refers to the RMSE variability among the 18 AWS sites. The circles indicate the mean values. The central lines in the box indicate the median, the box edges are drawn at the 25th (p_25_) and (p_75_) percentiles. The whiskers extend to the most extreme data values, after excluding the outliers. Values are considered as outliers if they are larger than p_75_ + 1.5(p_75_ − p_25_) or smaller than p_25_ − 1.5(p_75_ − p_25_).

Figure 4 shows the impact of the forecast errors of the individual weather variables on the RMSE of the forecasted daily ET_c-PM._ RMSE is computed at each station for each of the five lead times, and then averaged between the five lead times. The first two box plots describe the RMSE of ET_c-PM_ computed with all variables produced by the weather forecasts (i.e., no observed weather variables were used for the RMSE assessment), in the year 2014 (light blue) and year 2015 (dark blue). The other couples of box plots indicate the RMSE of ET_c-PM_ after substituting one forecast variable with the corresponding ground sensor measurement, while using the weather forecasts for the other variables. 

The largest RMSE reduction is observed when the ground sensor data are used to substitute the relative humidity, i.e., the largest impact is due to errors in the forecasted relative humidity, which affects the vapor pressure deficit of the air relevant for the aerodynamic component of the Penman–Monteith equation.

### 3.2. Calibration and Validation of the Hargreaves–Samani Equation

The uncalibrated Hargreaves–Samani equation overestimates the reference evapotranspiration at most AWS sites, i.e., ET_0-HS,obs_ computed with Equation (4) for *k_HS_* = 1 tends to be larger than ET_0-PM,obs._ As a result (see Table 2), the calibrated *k_HS_* is smaller than 1 at all AWS sites, except for AWS n. 5 and 10, where wind speed is generally very high, and thus the aerodynamic term (neglected by the Hargreaves–Samani equation) is more important. The average *k_HS_* is 0.85, ranging from 0.64 to 1.14. RMSE in the calibration period ranges from 0.51 to 1.04 mm/day, with an average value of 0.69 mm/day, while from 0.54 to 1.25 mm/day in the validation period, with an average value of 0.78 mm/h. Similar results were achieved by previous studies [29]. 

**Figure 5:**
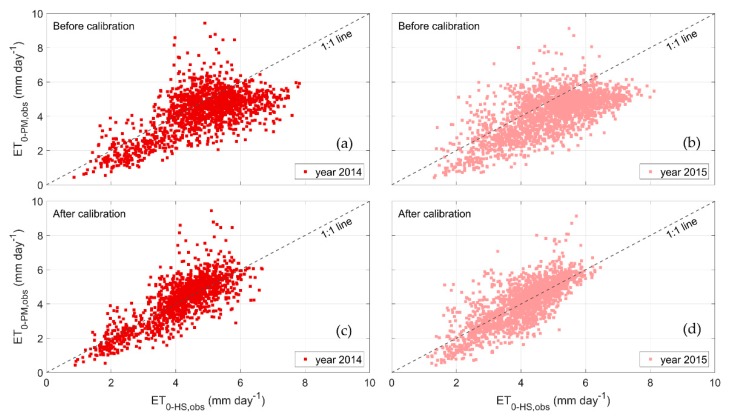
Scatter plots of ET_0-PM,obs_ versus ET_0-HS,obs_ before (**a**,**b**) and after calibration (**c**,**d**): (**a**–**c**) year 2104; (**b**–**d**) year 2105.

Figure 5 shows the scatter plot of the ET_0-PM,obs_ versus ET_0-HS,obs_ before and after the calibration applied to the validation dataset, of all AWSs in year 2014 and 2015. The scatter plots clearly show that the calibration of the *k_HS_* coefficient reduces both the bias and the spread of the predicted ET_0-HS,obs_ compared with ET_0-PM,obs_.

### 3.3. Remotely Assessed Crop Coefficients

**Figure 6:**
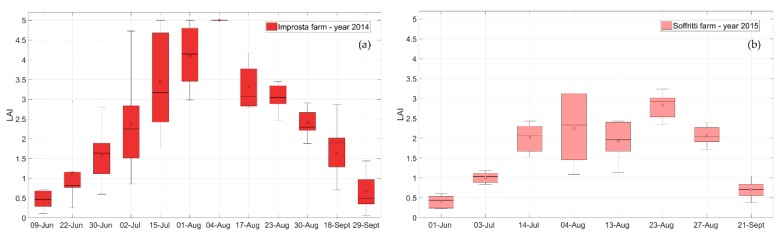
The leaf area index (LAI) at (**a**) Improsta and (**b**) Soffritti farms retrieved from remote multispectral satellite images.

LAI was the crop canopy parameter that mostly influenced the temporal pattern of the crop coefficient computed according to Equation (8). Figure 6 shows the box plots of the LAI retrieved at the reference maize fields. The variability of the LAI observed at each date reflects the spatial variability of maize growth and biomass accumulation among the different selected fields. Maize at Improsta is representative of the expected temporal pattern of a maize canopy in favorable weather conditions in Southern Italy, reaching the maximum value (5) at flowering at the beginning of August. LAI patterns observed at Soffritti were affected by the anomalous weather of the summer in the year 2015, which determined less vigorous development of the maize crop. 

These two data sets of Improsta and Soffritti were selected for evaluating the suggested procedure, as they are representative of two extremely different crop conditions, and because they were favorably validated with field LAI observations [6].

Albedo also changes along with the crop development, from 0.15 in the early stage of development to 0.20 at the maximum development, but with a less pronounced variability among the different sites within the same farm and years.

**Figure 7:**
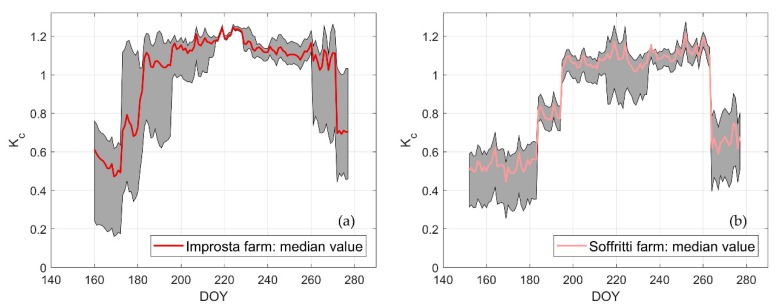
Temporal pattern of *K_c_* at (**a**) Improsta and (**b**) Soffritti farms. The gray band reflects the effect of LAI variability at each farm.

Figure 7 shows an example of the temporal pattern of the crop coefficient *K_c_* at the two farms computed with weather data observed at AWS n. 16. The gray band reflects the effect of the LAI variability at each farm, as depicted by the box plots in Figure 6. The crop coefficient in the early stage of development is around 0.5. In the mid-season it reaches 1.2 at Improsta and 1.1 at Soffritti. 

### 3.4. Forecasts of Crop Evapotranspiration

The performance of the ET_c_ forecasts was evaluated with an ensemble data obtained by combining observed and forecasted weather data at 18 sites, and the ensemble of crop canopy collected from multispectral images in year 2014 and 2015. 

Two methods were compared:

- ET_c-PM_, crop evapotranspiration forecasted by exploiting NWP forecasts for the entire set of weather variables required by the Penman–Monteith equation;
- ET_c-HS_, crop evapotranspiration forecasted by exploiting NWP forecasts only for the air temperature, according to the proposed methodology.

The benchmark is represented by (ET_c-PM,obs_), i.e., the crop evapotranspiration estimated with Equations (5)–(7) by using weather input data retrieved from ground sensors data according to the one-step procedure [3,4].

The forecast performances were evaluated by analyzing ET_c_ accumulated in 1, 3 and 5 days after the forecasts were issued, since the interest was to evaluate the error in the estimated accumulated crop water requirement, which is relevant for scheduling the irrigation with different time intervals.

**Figure 8:**
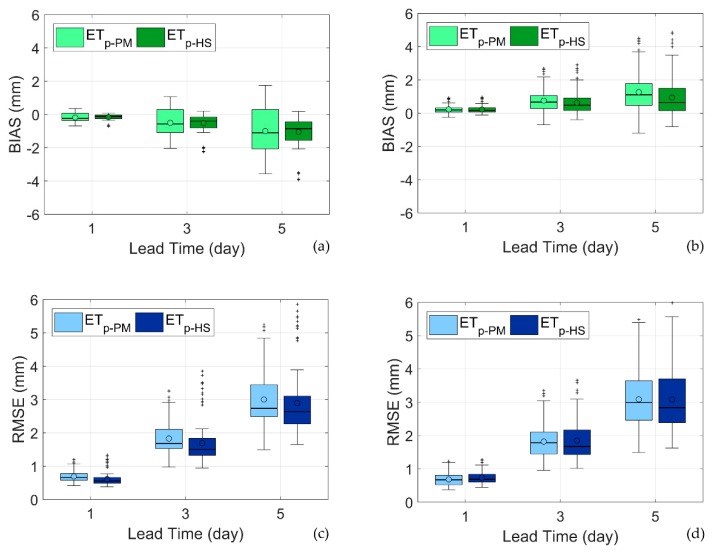
(**a**,**b**) BIAS (mm) and (**c**,**d**) RMSE (mm) of forecasted ET_c_ accumulated in 1, 3 and 5 days for increasing lead time with reference to year 2014 (**a**–**c**) and year 2015 (**b**–**d**). The spread of the box plots refers to the variability of the statistical indices among the 18 AWS sites. The circles indicate the mean values. The central lines in the box indicate the median, the box edges are drawn at the 25th (p_25_) and (p_75_) percentiles. The whiskers extend to the most extreme data values, after excluding the outliers. Values are considered as outliers if they arelarger than p_75_ + 1.5(p_75_ − p_25_) or smaller than p_25_ − 1.5(p_75_ − p_25_).

Figure 8 presents the boxplots of the BIAS and RMSE in years 2014 and 2015, computed with the ensemble of scenarios, obtained by combining the crop parameters at the Improsta and Soffritti farms, with observed and forecasted weather variables at the 18 sites. 

The accumulated forecasts underestimate ET_c_ in 2014, while they overestimate ET_c_ in 2015. In absolute terms, the median BIAS is around 0.2 for the first day of forecast, and accumulates up to 1 mm at the 5^th^ day of forecast (i.e., almost 0.2 mm/day). ET_c-HS_ BIAS is slightly smaller than ET_c-PM_ in absolute terms. More interestingly, the spread of the ET_c-PM_ BIAS is larger than the ET_c-HS_ BIAS, especially in year 2014, revealing the impact of the larger forecast uncertainty due to the larger number of weather variables involved. The results in year 2015 are affected by the anomalous summer weather conditions, with strong atmospheric instabilities generating thunderstorms and strong winds in the afternoon [30].

Median RMSE is around 0.75 mm for the first day of forecast, and increases up to around 2.75 mm at the 5^th^ day of the accumulated forecast. The RMSE in 2015 is generally larger than in 2014, due to the strong weather instabilities in the summer of 2015, as mentioned above, which were more challenging to be predicted by the NWP model. In all cases, the ET_c-PM_ median RMSE is larger than ET_c-HS_ median RMSE. As for the BIAS, in the year 2014, ET_c-PM_ exhibits an RMSE spread much larger than ET_c-HS_, as a result of the larger number of weather forecast variables involved in ET_c-PM_. The two spreads are comparable in the year 2015, since ET_c-HS_ predictions were more exposed to the unstable weather conditions of the summer of 2015.

Overall, the performance of ET_c-HS_ forecasts, based on temperature forecasts only, are comparable or even better than ET_c-PM_ forecasts, which instead require a larger set of forecast weather variables.

## 4. Discussion and Conclusions

Irrigation advisory services should be designed to meet the requirements of three different user categories [5]: farmers, water managers responsible for irrigation water allocation at the district level, and water management authorities. The first two categories require irrigation advices based upon forecasts of crop water requirements predicted days ahead, in order to allow the optimization of the water irrigation allocation and irrigation schedules, accounting for the actual crop water needs. In many irrigated areas, such as in the Southern Mediterranean countries, forecasting crop water requirements essentially means forecasting crop evapotranspiration, since rainfall and ground water contributions are negligible. A forecast horizon of at least 5–7 days is desirable from an operational perspective [4]. 

To this scope, two conditions are required:

- Crop canopy parameters need to be updated with relatively high frequency;
- Reliable weather forecasts should be available, possibly locally bias-corrected by means of ground weather observations.

These two conditions are met by the procedure proposed in this study, with the advantage of using just temperature as the forecast variable, as the input of the Hargreaves–Samani equation, instead of the entire set of weather variables required by the Penman–Monteith equation.

The recent ESA Sentinel-2 mission [31] opened the possibility to estimate crop parameters every 5 days with a resolution of 10 m with free multispectral images. The temporal evolution of the crop parameters within this time window is generally negligible, and thus up-to-date crop parameters can be assumed to be invariant within forecast horizons of 5–7 days [6,32]. Since this study refers to those years 2014–2015, it was not possible to use Sentinel-2 imagery for crop parameter estimates. However, by combining two different satellite imageries provided by remote sensors operating on DEIMOS-1 and LANDSAT 8 satellites, a temporal frequency of 7–15 days for updating data could be achieved. Moreover, the spectral images of these two satellites provided an effective reconstruction of the temporal pattern of the crop parameters, as confirmed by the field campaigns conducted in two experimental sites in Campania.

In this research, we used COSMO-LEPS forecasts, a Limited Area Model operating in Italy, and providing weather data at high spatial and temporal resolutions, with forecast horizon of 5–7 days. This category of NWP models is characterized by high reliability, but their forecasts are distributed at high costs of subscription [10]. We showed that these costs could be reduced by using a simplified ET_c_ estimation method, which employs only temperature forecasts.

There are a few reasons supporting the advisability of developing forecasting procedures that need only air temperature forecasts, rather than the entire set of weather variables affecting ET_c_ processes. The forecast performances are not homogenous, being highly dependent on the local terrain features. Air temperature is better predicted than other meteorological variables, since it is less affected by sub-grid variabilities. 

Post-processing techniques for correcting weather forecasts are more effective for air temperature than for other weather variables [20]. Differently from other weather variables, meteorological services freely broadcast numerical data of air temperature forecasts, by publishing them on dedicated web platforms. 

The methodology presented in this paper has been designed for those sites where reliable ground weather stations are available and the Hargreaves–Samani equation works reasonably well.

The procedure exploits the remote sensing-based Penman–Monteith direct approach (also known as one-step approach, [3]) for assessing analytically the current ET_c_ and crop coefficient. The crop coefficient approach is instead exploited for forecasting ET_c_ in combination with the Hargreaves–Samani equation.

The results at 18 sites across the Campania region have proven that the suggested procedure produces results that are as accurate and precise as those provided with a complete set of forecasted weather variables, with better performances at sites and in weather conditions where the radiative component of the ET_c_ equation is dominant over the aerodynamic term. In fact, in windy areas, the prediction performances of the Hargreaves–Samani equation were worse. In examined cases, for example, the highest root mean square errors were obtained at AWS sites n. 5-10-12, characterized by average wind speed larger than 4 m/s. 

The prediction performances in summer 2015 were clearly worse than in 2014 for a lead time greater than 1 day, since summer 2015 was characterized by anomalous thunderstorms. This circumstance mainly worsened the performance of temperature forecasts with lead time greater than 1 day, due to the inability of NWP models to predict the convective storms and to describe their small-scale variability. As a consequence, a simplified method based only on temperature forecasts for estimating ET_c_ resulted in being less accurate in predicting the evaporative demand of the atmosphere at sites affected by those thunderstorms. However, even in 2015, the proposed procedure produced prediction errors comparable with those obtained by implementing the Penman–Monteith equation.

In this perspective, possible improvements could be achieved by adopting more advanced correction procedures of both Hargreaves–Samani and of the forecasted air temperature. In this study, a simple bias correction was adopted for correcting air temperature forecast errors. However, more sophisticated procedures could be implemented, accounting for the non-stationarity of the forecasting error [20,33]. Similarly, the Hargreaves–Samani equation was calibrated by applying a scaling factor. However, other procedures could be also tested, by involving other constants of the Hargreaves–Samani equation into the calibration process [34,35].

## Figures and Tables

**Table 1:** List of the available Automatic Weather Stations (AWSs) with the average measured weather values in the period 2010–2015.

AWS	Elevation	Latitude	Longitude	T	WS	RH	RS
	(m a.s.l.)	(°)	(°)	(°C)	(ms^−1^)	(%)	(Wm^−2^)
1	848	40.65	14.54	20.3	2.1	72.3	243.3
2	631	41.2	15.14	21.3	3.0	67.6	264.1
3	236	41.12	14.83	22.8	1.9	68.5	262.8
4	9	41.2	13.84	23.4	1.9	77.1	265.3
5	770	40.86	15.28	20.8	4.4	69.5	267.9
6	1	40.94	14.02	23.7	2.5	76.7	268.5
7	515	40.84	15.04	20.2	1.2	71.5	251.7
8	552	40.26	15.66	20.7	1.5	77.0	256.9
9	88	40.79	14.16	25.7	3.2	74.9	276.3
10	660	40.56	15.24	21.4	4.0	66.8	256.4
11	62	41.43	13.88	23.4	1.2	76.0	240.3
12	750	41.42	15.04	20.6	4.2	70.3	266.4
13	31	41.02	14.34	25.8	1.9	67.1	255.9
14	167	41.25	14.47	23.7	1.9	70.9	260.2
15	13	40.64	14.84	26.0	2.1	65.5	261.6
16	413	40.13	15.46	23.2	2.6	71.5	260.8
17	117	41.34	14.33	22.9	1.1	67.0	254.4
18	64	40.61	14.98	24.7	1.3	63.3	263.7

**Table 2:** Calibrated *k_HS_* values along with RMSE for the calibration (2010–2013) and validation (2014–2015).

AWS	k_HS_	RMSE (mm day^−1^)	RMSE (mm day^−1^)	AWS	k_HS_	RMSE (mm day^−1^)	RMSE (mm day^−1^)
	(-)	Calibration Period	Validation Period	n.	(-)	Calibration Period	Validation Period
**1**	0.95	0.88	0.83	**10**	1.14	0.99	1.15
**2**	0.88	0.76	0.86	**11**	0.64	0.56	0.68
**3**	0.75	0.61	0.66	**12**	0.98	1.02	1.25
**4**	0.80	0.55	0.54	**13**	0.80	0.66	0.82
**5**	1.05	1.04	1.13	**14**	0.79	0.71	0.81
**6**	0.86	0.65	0.62	**15**	0.87	0.58	0.63
**7**	0.66	0.54	0.59	**16**	0.98	0.75	0.86
**8**	0.75	0.51	0.57	**17**	0.74	0.53	0.58
**9**	0.99	0.72	0.85	**18**	0.82	0.51	0.65
